# Molecular RNA Correlates of the SOFA Score in Patients with Sepsis

**DOI:** 10.3390/diagnostics11091649

**Published:** 2021-09-09

**Authors:** Agnes S. Meidert, Dominik Buschmann, Florian Brandes, Kristiyan Kanev, Jean-Noël Billaud, Melanie Borrmann, Matthias Witte, Benedikt Kirchner, Marlene Reithmair, Michael W. Pfaffl, Gustav Schelling

**Affiliations:** 1 Department of Anesthesiology, University Hospital, Ludwig-Maximilians-University Munich, 81377 Munich, Germany; dominikbuschmann@googlemail.com (D.B.); florian.brandes@med.uni-muenchen.de (F.B.); melanie.borrmann@med.uni-muenchen.de (M.B.); matthias.witte@med.uni-muenchen.de (M.W.); gustav.schelling@med.uni-muenchen.de (G.S.); 2 Division of Animal Physiology and Immunology, Technical University of Munich, 85354 Freising, Germany; kanev@wzw.tum.de (K.K.); benedikt.kirchner@wzw.tum.de (B.K.); michael.pfaffl@wzw.tum.de (M.W.P.); 3 QIAGEN Bioinformatics, Redwood City, CA 94063, USA; Jean-Noel.Billaud@qiagen.com; 4 Institute of Human Genetics, University Hospital, Ludwig-Maximilians-University Munich, 80336 Munich, Germany; marlene.reithmair@med.uni-muenchen.de

**Keywords:** organ dysfunction scores, high-throughput nucleotide sequencing, micro RNAs, messenger RNA, sepsis

## Abstract

The most common scoring system for critically ill patients is the Sequential Organ Failure Assessment (SOFA) score. Little is known about specific molecular signaling networks underlying the SOFA criteria. We characterized these networks and identified specific key regulatory molecules. We prospectively studied seven patients with sepsis and six controls with high-throughput RNA sequencing (RNAseq). Quantitative reverse transcription PCR (RT-qPCR) confirmation was performed in a second independent cohort. Differentially and significantly expressed miRNAs and their target mRNA transcripts were filtered for admission SOFA criteria and marker RNAs for the respective criteria identified. We bioinformatically constructed molecular signaling networks specifically reflecting these criteria followed by RT-qPCR confirmation of RNAs with important regulatory functions in the networks in the second cohort. RNAseq identified 82 miRNAs (45% upregulated) and 3254 mRNAs (50% upregulated) differentially expressed between sepsis patients and controls. Bioinformatic analysis characterized 6 miRNAs and 76 mRNA target transcripts specific for the SOFA criteria. RT-qPCR validated miRNA and mRNAs included IGFBP2 (respiratory system); MMP9 and PDE4B (nervous system); PPARG (cardiovascular system); AKR1B1, ANXA1, and LNC2/NGAL (acute kidney injury); GFER/ALR (liver); and miR-30c-3p (coagulopathy). There are specific canonical networks underlying the SOFA score. Key regulatory miRNA and mRNA transcripts support its biologic validity.

## 1. Introduction

Since its development in the 1990s, the Sequential Organ Failure Assessment (SOFA) score has become a standard instrument in the ICU to quantify acute morbidity of critically ill patients [1]. The SOFA score has been used in thousands of clinical investigations. Recent revisions in the definition of sepsis included SOFA as a key element [2] and the European Medicines Agency (EMA) has accepted the SOFA score as an endpoint in exploratory trials for sepsis.

SOFA is based on six different sub scores, one each for the respiratory, cardiovascular, hepatic, coagulation, renal, and neurological systems, which span from 0 to 4, and a final summary score is then calculated ranging from 0 to 24 points [3]. Increased scores reflect more severe organ dysfunctions [4].

Despite the widespread use and clinical validation of the SOFA score, it merely reflects a numeric simplification of morbidity. There appears to be a missing link between the SOFA criteria (e.g., the PaO2/FiO2 ratio for respiratory system or creatinine plasma concentrations for acute kidney injury) and the specific molecular signaling patterns underlying these criteria.

We addressed this issue by high-throughput and unbiased sequencing (RNAseq) of small non-coding miRNAs and protein-coding mRNAs from blood cells in patients with sepsis and healthy controls. For both types of RNA, transcripts significantly regulated between patients and controls were first identified by differential gene expression analyses. Bioinformatic methods were then applied to construct gene networks and to identify key regulatory molecules that specifically reflect the SOFA criteria from the RNAseq data set. We assumed that this novel approach could help to validate the score on a molecular level and may also be applied to the development of more refined scoring systems combining numeric indicators of organ dysfunctions with molecular findings.

## 2. Patients and Methods

For the initial characterization of SOFA-related molecular networks by RNAseq, we used blood cell-derived RNA in seven patients with septic shock and six healthy controls. mRNA sequencing was performed, while miRNA expression data were already available from this group [5]. We identified significantly regulated miRNAs and mRNAs and their interaction, filtered for SOFA criteria and constructed canonical networks. For RT-qPCR confirmation of selected miRNAs and mRNAs, a second group of patients with septic shock (*n* = 20) and healthy controls (*n* = 5) was then matched to the original RNAseq group from an independent and prospectively recruited sample of patients with septic shock (overall group size *n* = 54). Table S1 in the online supplement presents the demographic and clinical data of these patient groups.

Sepsis was diagnosed according to the 2016 Sepsis-3 definition [6] and using all available information including imaging, antibiotic response, and surgical findings; the final diagnosis of sepsis was made by experienced ICU clinicians without knowledge of the RNAseq results.

### 2.1. Calculation of the SOFA Score

SOFA calculation for the study was performed at admission to the ICU. Table 1 gives the SOFA criteria, their associated clinical conditions, and the bioinformatic correlates used in the study, and Table S2 in the online supplement presents the raw data used for SOFA score calculation in the RNAseq and the RT-qPCR confirmation group. When parameters for the SOFA criteria were below the minimal values for the criteria, 0 points were given [1]. In the case of values between two cut-off values for sub scores, the higher value was selected. When evaluating the SOFA component for central nervous dysfunction, we did not correct for the effects of sedative drugs, resulting in artificially lowered Glasgow Coma Scores. Instead, we included the sedative drugs propofol and midazolam into the network and identified possible target molecules of these compounds. Likewise, in the network reflecting the cardiovascular system, no conversion of angiotensin II units to the norepinephrine dose was performed as recommended [3], but their respective molecular targets were identified.

### 2.2. Time Points of Measurement, Sample Collection, and Patient Data

Blood sampling and recording of clinical data were performed at admittance to the ICU, along with the calculation of the admission SOFA. Details on blood sampling, sample collection, RNA processing, high-throughput sequencing, and RT-qPCR confirmation are given in the online supplement.

### 2.3. Inclusion and Exclusion Criteria of Patients

We included patients with sepsis fulfilling the following criteria: (1) age ≥ 18 years and (2) onset of signs of infection within the last 24 h severe enough to require ICU admission. Exclusion criteria were (1) pregnancy, (2) known infection by virus (e.g., human immunodeficiency virus), (3) malignancy, (4) pre-existing immunologic disorders (e.g., rheumatoid arthritis), (5) immunosuppressive medication (e.g., transplantation), and (6) severe pre-existing cardiovascular disorders.

### 2.4. Bioinformatic Analysis

Ingenuity pathway analysis (IPA, QIAGEN, Redwood City, CA, USA, Version 60467501) was used to identify functional interactions between the observed miRNAs, mRNAs, and protein functions.

For network analysis and identification of significant pathways in relation to the SOFA symptom criteria, we used the file containing all significantly regulated miRNAs between sepsis patients and controls and proceeded stepwise: (1) differentially expressed miRNAs were filtered to experimentally observed and findings in humans by the IPA microRNA target filter and (2) paired with the differentially expressed mRNAs from the mRNA data setting by IPA expression pairing to miRNA up to mRNA down, and vice versa (based on expression fold-change values). The resulting data set was then filtered to match SOFA criteria by selecting the compounds and conditions evaluated by SOFA (e.g., increase in creatinine) and the disorders typically associated with the respective criteria (e.g., for creatinine, acute kidney injury was selected) to increase specificity. Table 1 shows the SOFA criteria, their associated clinical conditions, and the bioinformatic filtering used for their operationalization. By this approach, we constructed molecular networks reflecting the respective SOFA criteria.

The overall disease state was set to “infectious disease” to exclude findings in cancer and other non-infectious inflammatory disorders. mRNAs identified as significantly expressed and targeted by miRNAs using the IPA microRNA target filter option and that were part of the above-mentioned signaling pathways were included using the IPA pathway function to create an interactome. Genes of interest and regulatory miRNAs were identified as a subset with direct interactions with other genes and miRNAs identified by the QIAGEN Knowledge Base and used for network construction [7].

Commonly used drugs with effects on SOFA criteria [3] and the sepsis mediator lipopolysaccharide (LPS) were included in the networks by selecting these compounds from the IPA genes and chemicals Knowledge Base, thus identifying their molecular targets (Table 1).

Specific regulation of miRNAs and mRNAs was confirmed in an independent cohort of 20 patients and 5 controls by RT-qPCR validation. The selection of miRNAs and mRNAs for confirmation was based on their regulatory function in the networks (miRNAs) and their possible targets (mRNAs) of the pharmacologic interventions (Table 1). Details on RT-qPCR sequencing are presented in the Supplementary Material.

### 2.5. Statistical Analysis

Demographic and clinical characteristics between controls and sepsis patients from both the RNAseq and the RT-qPCR group were compared using the Wilcoxon rank sum test for nonparametric continuous variables and the Chi-square or Fisher’s exact test for comparison of categorical variables. Statistical significance of RNA expression changes between sepsis patients and controls were assessed using Student’s *t*-test. Data in the text and in tables are reported as median and interquartile range. Statistical calculations were performed using SPSS (version 24.0, IBM, Armonk, NY, USA).

### 2.6. Ethics Approval and Patient Consent for Study Participation

The study was approved by the Ethics Committee of the Medical Faculty of the University of Munich (protocol #551-14) and carried out according to the World Medical Association Declaration of Helsinki. All study samples were pseudonymized during analysis.

Written informed consent for study participation including publication of blinded individual personal data were obtained from each participant or the patient’s legal representative.

## 3. Results

There were no significant differences in SOFA scores, biometric data, source of infection, or number and type of organ failure between patients from the RNAseq and the RT-qPCR confirmation groups. Table S1 in the Online Supplement gives these data and illustrates the results of the matching process between the two groups.

**Figure 1:**
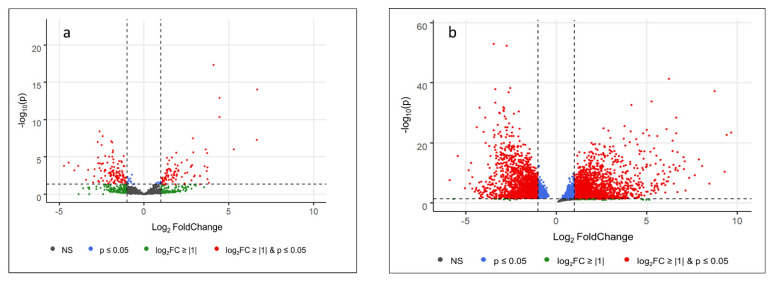
Volcano plots of miRNA (**a**) and mRNA (**b**) regulation from RNAseq. Dashed lines: significance thresholds for log2 fold change and *p*-value. NS: not significant; log2FC: log2 fold change.

Figure 1 shows the significance thresholds for log2 fold changes and *p*-values for miRNA and mRNA regulation from the RNAseq data set. Further detailed results of RNA characterization according to subgroups, differential gene expression analysis, and RT-qPCR validation of selected miRNAs and mRNAs are shown in the Supplementary Material in Tables S3–S8 and in the Figures S1 and S2 of the Supplement. Table S8 lists all significantly regulated RNAs unique to each of the respective SOFA criteria.

### 3.1. Networks for SOFA Criterion 1: Hypoxia

**Figure 2:**
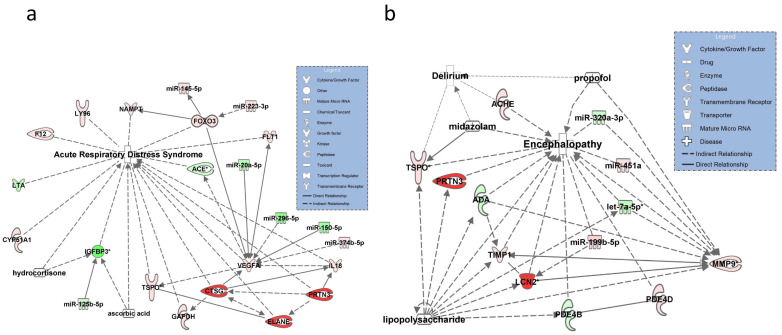
Molecular networks representing the SOFA symptom criteria 1 (“hypoxia”, left) and 2 “impaired Glasgow Coma Scale” (GCS, right). (**a**) The SOFA symptom criteria 1 was operationalized by the disease state acute respiratory distress syn-drome (ARDS) resulting in hypoxia. The network shows miRNAs and mRNA targets potentially involved in ARDS identified from the RNAseq and the RT-qPCR dataset. Hydrocortisone and ascorbic acid were included in the model targeting IGFB3 (insulin-like growth factor-binding protein 3). IGFB3, ACE (angiotensin-converting enzyme), and TSPO (mitochondrial permeability transition pore, also functioning a peripheral benzodiazepine receptor) were validated by RT-qPCR. (**b**) The molecular network for the SOFA symptom criteria 2 is visualized by the disease status septic enceph-alopathy and delirium. Let-7a-5p, miR-30c-5p, LNC2 (Lipocalin 2), MMP9 (matrix metallopeptidase 9), and TSPO were RT-qPCR validated in this network. Target transcripts of lipopolysaccharide and the sedatives propofol and midazolam, frequently used in patients with sepsis and resulting in lower GCS scores, are illustrated by arrows. Successfully validated molecules are marked with an asterisk in both networks. Solid lines connecting molecules in the networks indicate direct regulatory interactions and dashed lines indirect relationships between molecules and disease states. Downregulated and upregulated miRNAs and mRNAs are shown in green and red in both figures, respectively.

The resulting network for SOFA criteria 1 is shown in Figure 2a. Transcripts identified from the mRNAseq data set with a known regulatory function in ARDS included LTA, F12, LY96, NAMPT, TPSO, and CYR5A1. Other important regulatory molecules associated with ARDS in this pathway included upregulated VEGFA interacting with TPSO (validated) and targeted by miR-296-5p, miR-20a-5p, miR-150-5p, and downregulated IGFBP3, which was validated by RT-qPCR. IGFBP3 was also unique for this pathway and identified as pharmacologic target for hydrocortisone and ascorbic acid (the synonyms for all transcripts are given in the abbreviation list).

### 3.2. Networks for SOFA Criterion 2: Glasgow Coma Scale

Molecules identified for this criterion from the RNAseq dataset with a known or possible involvement in encephalopathy and delirium included miR-320a-3p, ACHE, TSPO, and TIMP1, which interacted with MMP9. Potential targets of LPS in the network included highly upregulated LNC2 (NGAL), MMP9, and TSPO (all validated by RT-qPCR). Upregulated MMP9 was targeted by miR-320-3p and let-7a-5p and was—along with PDE4B—unique for this pathway (Table S8). Propofol targeted MMP9 and Midazolam TSPO, both along with LPS (Figure 2b).

### 3.3. Network for SOFA Criterion 3: Circulatory Failure

**Figure 3:**
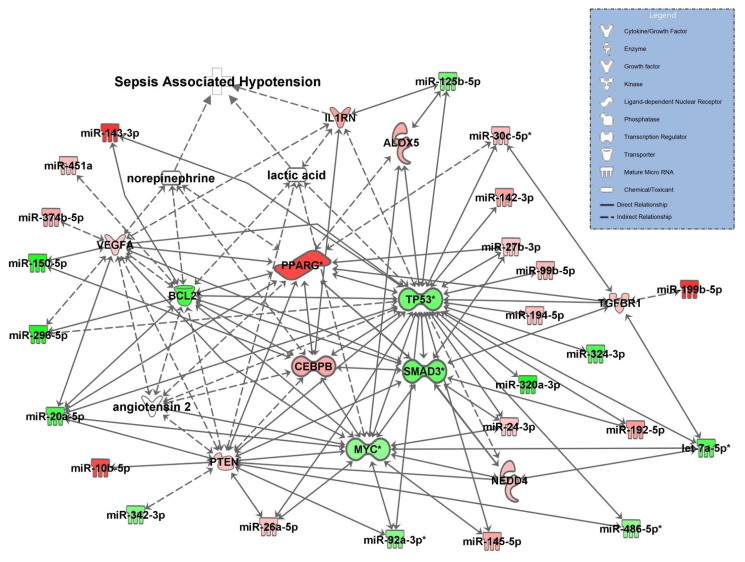
Molecular network for SOFA symptom criterion 3 “circulatory failure”, represented by the disease state sepsis associated hypotension and the network cardiovascular signaling in sepsis. let-7a-5p, miR-30c-5p, miR-486-5p, miR-92a-3p, BCL2 (BCL2 apoptosis regulator), MYC (bHLH transcription factor), PPARG (peroxisome proliferator-activated receptor gamma), SMAD3 (SMAD family member 3), and TP53 (transformation related protein 53) were RT-qPCR validated and are marked with an asterisk. Possible target molecules of the two vasopressors norepinephrine and angiotensin II are shown along with transcripts associated with increased lactate levels (s. text). Downregulated and upregulated miRNAs and mRNAs are marked green and red, respectively. Solid lines connecting molecules indicate direct regulatory interactions and dashed lines indirect relationships between molecules in the network.

The SOFA criterion 3 (Figure 3) reflecting circulatory failure was assessed by disease status hypotension and cardiovascular signaling in sepsis, and the vasopressors norepinephrine and angiotensin II and lactate concentrations were included in the network. As expected, the model demonstrated a direct relationship between norepinephrine and lactic acid, but also identified upregulated IL1RN from the mRNAseq dataset as directly involved with sepsis-associated hypotension. Lactate production in the pathway was also associated with IL1RN, targeted by miR-125-5p, PTEN, TP53, BCL2, and MYC. Norepinephrine and angiotensin II targeted VGFA, PPARG (validated), and CEBPB. Angiotensin II showed an association with TP53 and PPARG and norepinephrine was associated with BCL2. PPARG and TP53 were validated and not found in other pathways.

### 3.4. Networks for SOFA Criterion 4: Increase in Serum Creatinine

**Figure 4:**
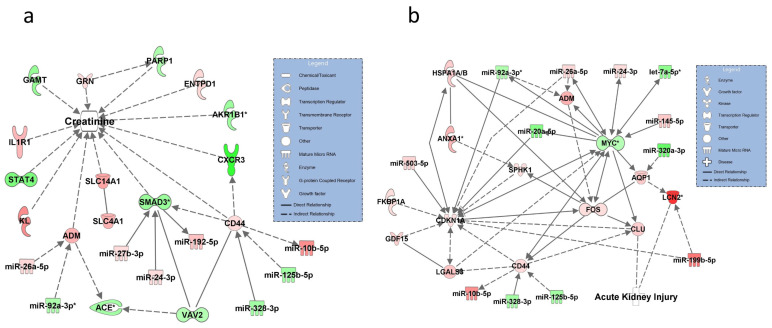
Molecular networks for the SOFA symptom criterion 4 “increase in serum creatinine” as visualized by the symptom increase in creatinine (**a**) and the disease state acute renal injury (**b**). miRNA-92a-3p, ACE (angiotensin converting enzyme), AKR1B1 (aldose reductase), SMAD3 (SMAD family member 3), LNC2 (lipocalin 2, NGAL), ANXA1 (Annexin1), MYC (bHLH transcription factor), let-7a-5p, and miR-92a-3p were validated by RT-qPCR in the networks and are marked with asterisks. Solid lines connecting molecules in the networks indicate direct regulatory interactions and dashed lines illustrate indirect relationships between molecules and creatinine (**a**) or disease states (**b**). Downregulated and upregulated miRNAs and mRNAs are marked in green and red, respectively.

For this criterion, two networks were constructed with the disease status set at increase in serum creatinine (Figure 4a) and acute renal injury in sepsis (AKI) (Figure 4b). In the first network, molecules in the pathway showing an association with serum creatinine included ACE (validated), interacting with upregulated ADM and regulated by miR-92a-3p and SMAD3 (validated) and targeted by miR-24-3p, miR-192-5p, and miR-27p-3p. AKR1B1 (validated) and KL were also related to creatinine levels (Figure 4a). The second network reflecting AKI centered around MYC (validated), regulated by miR-24-3p, miR-20a-5p, and miR-145-5p, interacting with ADM, was targeted by the validated miR-92a-3p. ANXA1 was validated and only found in this pathway. The model identified a regulatory sub-pathway leading from ANXA1 to SPHK1 to FOS to CLU, which was directly related to AKI (Figure 4b). A second molecule with a direct relationship to AKI was LNC2 (validated), interacting with AQP1 and regulated by MYC.

### 3.5. Networks for SOFA Criterion 5: Increase in Bilirubin

**Figure 5:**
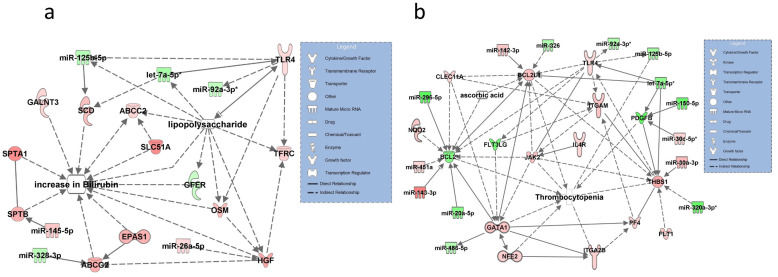
Molecular networks for SOFA symptom criteria 5 “increase in bilirubin” visualized by the disease state “septic liver failure” (**a**) and the SOFA symptom criteria 6 “low platelet count” (**b**). Possible effects of lipopolysaccharide are shown in Figure 5a with arrows pointing at target transcripts. let-7a-5p and miR-92a-3p were validated by RT-qPCR in criteria 5 and let-7a-5p, miR-30c-5p, miR-486-5p, and miR-92a-3p in criteria 6 and are marked with an asterisk. Downregulated and upregulated miRNAs and mRNAs are marked in green and red, respectively. Solid lines connecting molecules in the networks indicate direct regulatory interactions and dashed lines illustrate indirect relationships between molecules and the SOFA item increase in bilirubin (**a**) or thrombocytopenia (**b**).

Significantly upregulated transcripts in this network were TLR4, which was activated by LPS and by the downregulated and validated miRNA let-7a-5p. Upregulated TLR4 was also shown to interact with the validated and downregulated miR-92-3p and with miR-125-5p targeting SCID. SCID was shown in the network to have a direct effect on bilirubin. Other upregulated transcripts in the network were ABCG2 and EPAS1; both had a regulatory function on bilirubin plasma levels (Figure 5a). No interactions were found for thiamin or ascorbic acid.

### 3.6. Networks for SOFA Criterion 6: Low Platelet Count

Upregulated transcripts identified in the network included PF4 indirectly targeting GATA 1 with a direct relationship to thrombopenia. Downregulated and validated BCL2 was also directly associated with thrombopenia. Upregulated miR-30c-5p (validated) targeted downregulated PDGFB. Other molecules directly associated in the network with thrombopenia included THBS1 (upregulated and targeted by the validated miR-320a-3p), ITGA 2b, and JAK2, all unique to this pathway (Figure 5b).

## 4. Discussion

In this study, we used RNAseq data and bioinformatic analysis to identify key regulatory molecules and constructed signaling networks to characterize molecular mechanisms underlying the clinically validated SOFA criteria.

When applying this approach to SOFA criterion 1 reflecting hypoxia (low PaO2/FiO2 ratio), we selected the disease state ARDS to increase network specificity, as hypoxia is also seen in other conditions (e.g., high altitude). A unique molecule for this criterion was downregulated and validated IGFBP3 with a direct relationship in the network to ARDS. The IGFBP3 pathway was involved in acute lung injury and ARDS in a proteomics study [8] and in fibrotic lung disease in ARDS. Lower plasma levels of IGFBP3 are associated with more severe ARDS and increased mortality [9]. Another interesting finding was the activation of the miR-296-5p, miR-20a-5p, miR-150-5p, and VEGFA pathway. Hypoxia is known as a potent inducer of VEGFA mRNA expression; however, regulatory effects of these miRNAs have not been described before.

One major target identified in the network of SOFA criterion 2 (Glasgow Coma Scale) was the highly upregulated transporter molecule LCN2. Interestingly, LCN2 has been characterized as the most substantially elevated protein in the brain of mice after an LPS challenge and showed neuroprotective effects during systemic inflammation [10]. The model also demonstrated binding of LCN2 to MMP9 [11], a collagen-degrading enzyme known to disrupt the blood–brain barrier and a pharmacologic target of propofol. Propofol is known to decrease the expression of the MMP9 protein [12,13] and the model suggests protective effects regarding neuroinflammation and the development of septic encephalopathy in the ICU. The benzodiazepine midazolam targeted TSPO, a peripheral benzodiazepine receptor [14], also activated by LPS in the model. Midazolam is known to suppress the LPS-stimulated immune responses of human macrophages via TSPO signaling [15], but the model did not identify possible neuroprotective pathways for this agent.

Interestingly, the translocator protein TSPO was also significantly regulated in the hypoxia pathway of SOFA criteria 1. TSPO is mainly found on the outer mitochondrial membrane [16] and is involved in mitochondrial permeability transition under hypoxic conditions [17], along with its abovementioned function as a peripheral benzodiazepine receptor. This points to a role of TSPO associated mitochondrial dysfunction in both pathways.

When constructing the signaling network for SOFA criterion 3, reflecting circulatory failure, upregulated IL1RN was identified as directly involved in hypotension and septic shock and in the production of lactic acid, indicating tissue hypoperfusion. Upregulation of IL1RN protein levels was described in patients with sepsis [18] and septic shock [19]. IL1RN was also tested in an unsuccessful clinical trial as a therapeutic agent for sepsis [20]. PPARG, a transcription factor of the nuclear hormone receptor superfamily, and TP53 were found to be unique for SOFA criterion 3. Genetic variations in the PPARG gene were linked to the outcome of sepsis and PPARG was suggested as a novel biomarker for sepsis [21]. The PPAR family exerts anti-inflammatory activities in immune cells, vascular smooth muscle cells, and monocytes [22]. Angiotensin II, a vasopressor used in refractory septic shock [23], inhibits PPARG activity, thereby increasing blood pressure [24].

The networks for SOFA criterion 4 (renal failure) identified numerous molecules. Among these, PARP1 appeared to have protective renal effects in knock-out mouse models of ischemic injury [25]. In contrast to PPAR1, IL1R1 (interleukin receptor 1) resulted in lower creatinine blood concentrations in models of ischemic injury [26].

The network reflecting AKI centered around MYC. Its central role as a regulator of gene expression was demonstrated in a pathway study comparing high-quality microarray studies of gene expression in AKI [27]. MYC also indirectly targets LNC2 (NGAL, validated and massively upregulated), a biomarker of renal injury regarded as originating from renal tubular cells. However, the protein is expressed and secreted by immune cells during systemic inflammation, which explains that LNC2 transcript can be detected in blood cells [28,29]. The pathway also suggests that the effect of downregulated MYC on LNC2 is mediated by an upregulation of AQP1, which has been confirmed as a MYC responsive gene in a microarray analysis [30].

The network representing an increase in bilirubin showed a direct effect of LPS on bilirubin in experimental animals [31]. GFER, also targeted by LPS, influences bilirubin levels in humans with liver disorders [32]. Serum GFER increased in a sepsis model after administration of LPS in rats and is mainly released from hepatocytes indicating hepatocellular stress [33], whereas in our network, resulting from RNAseq of blood cells, GFER appeared to be downregulated, suggesting tissue specific effects in GFER mRNA expression.

The complex network constructed for SOFA criterion 6 (low platelet count) included downregulated PDGFB signaling by the upregulated miR-30c-5p (PDGFB knock-out mice showed thrombocytopenia [34]). The model also identified an association between GATA1 and increased expression of PF4 [35]. PF4 plays an important role in development of sepsis induced thrombocytopenia [36].

The major aim of our study was to construct signaling networks directly related to SOFA sub scores using high-throughput RNAseq in patients with sepsis. We detected many significantly regulated RNAs with several miRNA and mRNA transcripts specific to the respective criteria. For construction of the networks, we used a broad bioinformatic approach. This methodology is unbiased, but often includes cellular and animal studies not primarily related to sepsis, but identified experimentally proven signaling networks only potentially underlying the SOFA sub scores. Furthermore, RNAseq was performed in RNA isolated from blood cells, whereas many molecules identified in the networks were investigated in different cell models or organs, but their presence in blood cells was confirmed by our sequencing data, a limitation that applies to almost all biomarkers used in critically ill patients.

Given the pronounced impact on the immunologic response to sepsis, one may have expected a much higher number of regulated molecules including interleukins and chemokine transcripts in each network reflecting any given SOFA sub score. The reason for the relatively few regulated molecules in our analysis is stringent filtering to clinical conditions and the most important bioinformatic correlate to the SOFA criteria under study (e.g., criteria 1—low PaO2/FiO2—was operationalized by hypoxia and, as a correlate, pulmonary capillary leak syndrome was selected). In addition, only molecules with an experimentally confirmed relationship to the SOFA context were included in the networks in order to focus on the most important and scientifically robust signaling pathways. This resulted in a further reduction of the number of regulated molecules in the networks and made their graphical representation clearer.

## 5. Conclusions

Our study identified specific signaling networks underlying the SOFA score and provides evidence for the biologic validity of this scoring system both for the assessment of critically ill patients and as inclusion criteria or endpoint for clinical studies. Further and more refined ICU disease severity systems could include expression values of key molecules identified in our study to increase their validity for the identification of patients with an increased risk for negative outcomes, direct therapeutical decisions, and lead to novel endpoints in sepsis trials.

## Figures and Tables

**Table 1:** SOFA criteria, their selected clinical correlates, and the bioinformatic filtering to disease states used for the construction of molecular networks from RNAseq data and for identification of target molecules of pharmacologic intervention.

SOFA Criteria	Clinical Condition	Bioinformatic Correlate	Selected Pharmacologic Intervention (Drugs and Compounds)	Canonical Pathway (Figure)
PaO2/FiO2 ratio	Hypoxia	Pulmonary capillary leak syndrome, ARDS^2^	Ascorbic acid, hydrocortisone	Figure 2a
Glasgow Coma Scale	Impaired cognition	Encephalopathy, delirium	Midazolam, propofol, lipopolysaccharide	Figure 2b
Mean arterial blood pressure	Hypotension	Cardiovascular signaling in sepsis	Norepinephrine, angiotensin II, lactic acide	Figure 3
Vasopressor administration	Circulatory failure	Norepinephrine, angiotensin II, lactate		
Creatinine	Acute kidney injury	Increase in serum creatinine, acute kidney injury		Figure 4a,b
Bilirubin	Acute liver failure	Increase in bilirubin	Lipopolysaccharide	Figure 5a
Platelet count	Coagulopathy	Low platelet count	Ascorbic acid	Figure 5b

## Data Availability

The data presented in this study are available on request from the corresponding author. The data will be publicly available in a data repository.

## Supplementary Materials

The following are available online at https://www.mdpi.com/article/10.3390/diagnostics11091649/s1.

## Abbreviations

ABCG2	ATP-binding cassette super-family G member 2
ACE	angiotensin converting enzyme
ACHE	acetylcholinesterase
ADM	adrenomedullin
AKR1B1	aldose reductase
ANXA1	lipocortin I
AQP1	aquaporin 1
BCL2	apoptosis regulator
CEBPB	nuclear factor of IL-6 expression
CLU	clusterin
CYR5A1	nicotinamide phosphoribosyltransferase
EPAS1	hypoxia-inducible factor-2 alpha
F12	coagulation factor XII
FOS	fos proto-oncogene, AP-1 transcription factor subunit
GATA 1	GATA binding protein 1
IGFBP3	insulin like growth factor binding protein 3
IL1RN	interleukin 1 receptor antagonist
ITGA 2b	integrin alpha 2B
JAK2	janus kinase 2
KL	Klotho
LNC2	lipocalin 2, NGAL
LTA	Lymphotoxin alpha
LY96	lymphocyte antigen 96
MMP9	matrix metalloproteinase-9
MYC	bHLH transcription factor
NAMPT	nicotinamide phosphoribosyltransferase
PDE4B	cAMP-specific 3′,5′-cyclic phosphodiesterase 4B
PDGFB	platelet derived growth factor subunit B
PF4	platelet factor 4
PPARG	peroxisome proliferator activated receptor gamma
PTEN	phosphatase and tensin homolog
SCID	stearoyl-CoA desaturase
SMAD3	SMAD family 3
SPHK1	sphingosine kinase 1
THBS1	thrombospondin 1
TIMP1	TIMP metallopeptidase inhibitor 1
TLR4	toll-like receptor 4
TP53	tumor protein 53
TSPO	translocator protein/peripheral-type benzodiazepine receptor
EGFA	vascular endothelial growth factor A
VGFA	vascular growth factor A
