# Supplementary material for: Molecular RNA Correlates of the SOFA Score in Patients with Sepsis
Source: Diagnostics (Basel). 2021 Sep 9;11(9):1649. doi: 10.3390/diagnostics11091649 (PMC8468706; doi:10.3390/diagnostics11091649)
Supplement: Supplementary file 1 [file diagnostics-11-01649-s001.zip › diagnostics-1336375-supplementary.pdf]

Molecular RNA Correlates of the SOFA Score in Patients with Sepsis

Agnes S. Meidert[1*], Dominik Buschmann[1/2*], Florian Brandes[1], Kristiyan Kanev[2], Jean-Noël Billaud[3], Melanie Borrmann[1], Matthias Witte[1], Marlene Reithmair[4], Michael W. Pfaffl[2], Gustav Schelling[1]
[1] Department of Anesthesiology, University Hospital, Ludwig-Maximilians-University Munich, Munich, Germany
[2] Division of Animal Physiology and Immunology, Technical University of Munich, Freising, Germany
[3]QIAGEN Bioinformatics, Redwood City, USA
[4] Institute of Human Genetics, University Hospital, Ludwig-Maximilians-University Munich, Munich Germany
*Both authors contributed equally to this work

Supplementary Data

Additional Methods

Details on sample collection

Blood samples were drawn from patients through intravascular catheters and from healthy subjects via venipuncture. A previous study from our group showed only trivial differences between extracellular RNA profiles in arterial and venous blood samples, suggesting that these sampling methods can justifiably be compared (1). Whole blood samples designated for extraction of cellular RNA were collected in RNA tubes (PAXgene, Qiagen, Hildesheim, Germany). RNA tubes were kept at room temperature for 24 h and stored at -80 °C according to the manufacturer`s protocol.

RNA isolation

Total RNA was extracted using the PAXgene Blood miRNA Kit (Qiagen, Hildesheim, Germany) according to the manufacturer's "manual purification" protocol. RNA was eluted

in 40 µl and immediately stored at -80 °C. RNA was quantified by capillary electrophoresis using the 2100 Bioanalyzer and the RNA 6000 Nano assay (Agilent Technologies).

High-throughput sequencing

Small RNA sequencing libraries were constructed as previously reported (2). Briefly, 300 ng total RNA were adaptor-ligated, reverse transcribed, barcoded and amplified using the NEBNext Multiplex Small RNA Library Prep Set for Illumina (New England BioLabs, Ipswich, MA, USA). Libraries were subsequently size-fractionated by high-performance gel electrophoresis, purified and assessed by capillary electrophoresis (High Sensitivity DNA Assay, 2100 Bioanalyzer, Agilent Technologies, Waldbronn, Germany),

Libraries for mRNA sequencing were constructed from 100 ng total RNA. Briefly, mRNA was captured by Poly(A) enrichment (NEBNext Poly(A) mRNA Magnetic Isolation Module, New England BioLabs) prior to library preparation using the NEBNext Ultra RNA Library Preparation Kit (New England BioLabs). Libraries were enriched and indexed by 13 cycles of PCR amplification using the NEBNext Dual Index Primers Set 1 (New England BioLabs). The quality of final libraries was assessed by capillary electrophoresis using the Agilent High Sensitivity DNA Kit (Agilent Technologies). Finally, libraries were quantified via qPCR using the KAPA SYBR FAST qPCR Master Mix (Kapa Biosystems, Wilmington, MA, USA).

Both miRNAs and mRNAs were sequenced using 50 cycles of single-end sequencing on a HiSeq 2500 (Illumina, San Diego, CA, USA).

High-throughput sequencing data analysis

Small RNA sequencing data were processes as detailed elsewhere (2). FASTQ files from mRNA sequencing were imported directly into the Array Studio software v10.0.1.81 (QIAGEN, Cary, NC, USA) package for further data analysis. All FASTQ files were aligned to

the Human B38 consensus and gene model Ensembl.v88 using the proprietary OmicSoft Aligner OSA (3).

Differential expression of miRNAs and mRNAs between patients and controls was assessed using DESeq2 (4). For miRNAs, a |log2 fold change| ≥ 1, an adjusted p-value (padj) of ≤ 0.05 and a mean expression (baseMean) of ≥ 50 reads were set as thresholds to identify significantly regulated transcripts. Thresholds for mRNA were set to |log2 fold change| ≥ |1, adjusted p-value (padj) ≤ 0.05 and baseMean ≥ 10 reads.

RT-qPCR validation

The SOFA criteria specific regulation of miRNAs and mRNAs was confirmed in a second step in an independent cohort of 20 sepsis patients and 5 healthy controls using RT-qPCR. The selection of miRNAs and mRNAs for RT-qPCR confirmation was based on their important regulatory function in the identified network (miRNAs) and their possible targets (mRNAs) of the pharmacologic intervention (s. Table 1 in the main manuscript).

For RT-qPCR confirmation, 10 ng of the selected miRNAs were reverse transcribed using the miRCURY LNA RT Kit (Qiagen) and diluted 1:60 as per the manufacturer's instructions. Expression of miRNAs was quantified via qPCR (miRCURY LNA SYBR Green PCR Kit, Qiagen) in a 20 µl reaction volume.

To validate mRNA expression, 850 ng total RNA was reverse transcribed using the QuantiTect Reverse Transcription Kit (Qiagen). qPCR was carried out on 20 ng cDNA using the SsoAdvanced Universal SYBR Supermix and pre-validated PrimePCR Assays (both Bio-Rad, Munich, Germany) in a 20 µl reaction volume. All qPCRs were run on MiniOpticon cycler (Bio-Rad) using the manufacturer's recommended thermal profiles. For both miRNA and mRNA, potential reference transcripts were selected from RNAseq data by geNorm and NormFinder (7). Upon testing the suitability of reference candidates in RT-qPCR, miRNAs and mRNAs

were normalized to the geometric mean of miR-501-3p, miR-625-3p and miR-181a-2-3p or Ubiquitin C (UBC), Ribophorin I (RPN) and Transmembrane BAX inhibitor motif containing 6 (TMBIM6), respectively. Relative quantification was performed using the ΔΔCq method (4).

Additional Results

RNA characterization

Total RNA was extracted from blood cells, yielding concentrations of 231.71 ± 173.25 ng/µl and 149.17 ± 44.90 ng/µl for sepsis patients and controls, respectively (Table S3). Small RNA and protein-coding RNA were profiled by RNAseq as described above. Boasting median Phred scores of 37 (33 – 38) (patients) and 37 (29-38) (controls), sequencing data for miRNAs were of excellent quality (Figure S1a). For mRNAs median Phred scores were 40 (38 – 40) for patients and 40 (38-40) for controls (Figure S1b).

Similar to initial RNA concentrations, the numbers of both miRNA and mRNA reads tended to be higher in patients compared to controls without reaching statistical significance for any (Table S3).

Next, we assessed the number of unique transcripts identified across study populations. Even though a small proportion of both miRNAs and mRNAs was exclusively identified in patients or controls, there was substantial overlap between the two groups (Table S4).

Differential gene expression

Expression profiles of miRNA and mRNA transcripts were assessed using DESeq2. Based on the criteria outlined above, a total of 82 miRNAs (37 up in patients; 45.12 %) were identified to be significantly regulated between patients and controls in the earlier study (Figure S2A) (8).

Log2 fold changes ranged from 1.01 to 6.68 and from -1.06 to -4.71 for significantly up- and downregulated miRNAs, respectively. Similarly, the abundance of 3,254 mRNAs (1,621 up in patients; 49.82 %) was significantly altered between study populations with log2 fold changes ranging from 1.00 to 9.67 and from -1.00 to -5.46 (Figure S2A). The most prominently up- and downregulated transcripts are reported in Table S5. In Principal Component Analysis (PCA), patients and controls were clearly separated for both miRNAs and mRNAs (Figure S2A and B).

RT-qPCR validation of selected miRNAs and mRNAs from the constructed networks

To verify conclusions drawn based on RNAseq data, six miRNAs and 30 mRNAs were selected for validation by RT-qPCR in the different and independent patient cohort. Five miRNAs could be validated by qPCR. Of the 30 *in silico* identified target mRNAs 13 could be validated in the same regulatory direction as in RNAseq. Individual results for each validated miRNA and mRNA are reported in

Table **S6**.

Additional Figures

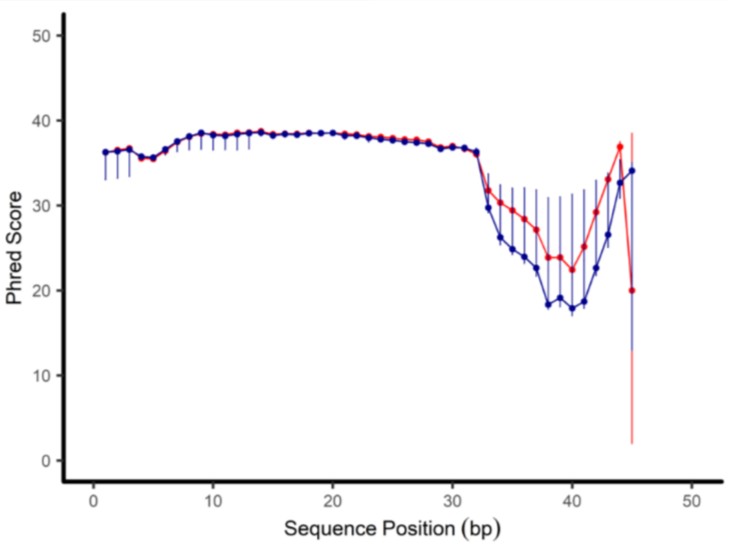

Figure S1a: Assessment of sequencing quality for small RNA-Seq. Samples from both sepsis patients (red) and volunteers (blue) featured Phred scores > 30 for the first 30 sequenced nucleotides.

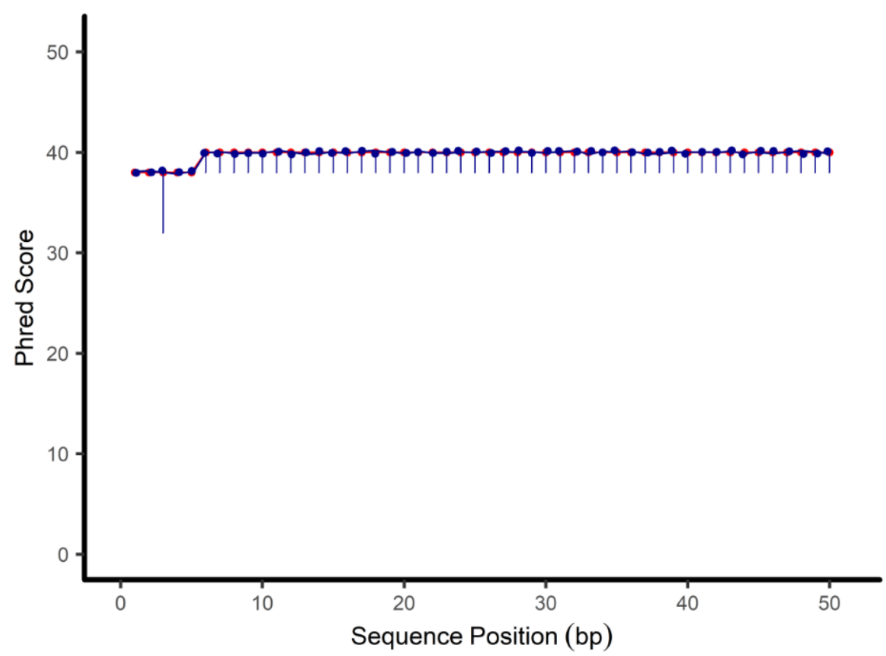

Figure S1b: Sequencing quality for mRNAseq samples from both sepsis patients (red) and volunteers (blue) showing Phred scores > 30 for the first 30 sequenced nucleotides.

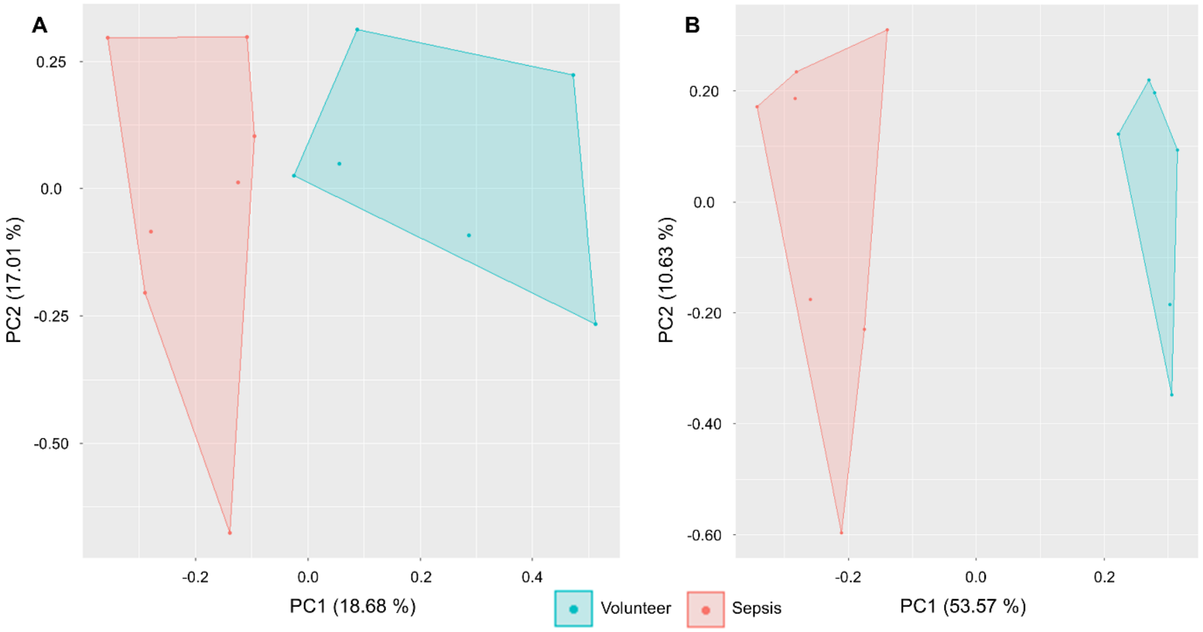

Figure S2: Comparison of miRNA (A) and mRNA (B) expression between sepsis patients and healthy volunteers by principal component analysis.

Additional Tables

Table S1: Demographic and clinical data of the study groups.

**Variable**	**Sepsis - NGS (n=7)**	**Sepsis – miRNA confirmation (n=20)**	**p-value[a]**	**Healthy volunteers NGS (n=6)**	**Healthy volunteers miRNA confirmation (n=5)[c]**	**p-value[b]**
Male/female (n)	6/1	12 / 8	0.438	4/2	3 / 2	0.689
Age (years)	58 (54 – 58)	61.5(54.2 - 67.0)	0.142	52 (48 – 58.2)	54.0 (51.0 – 55.0)	0.784
Weight (kg)	98 (72.5 – 100)	81.0(69.2 - 102.5)	0.719	70.5 (66.5 – 92.5) (p=0.26)	80.0 (76.0 – 82.0)	0.361
Height (cm)	179 (172.5 – 182.5)	172.5 (166.5 - 178.5)	0.279	165 (162.8 – 173.2) (p=0.08)	181.0 (174.0 – 183.0)	0.142
Body mass index (BMI)	28.3 (27.7 – 29.9)	26.1 (23.6 – 31.8)	0.782	26.4 (24.4 – 27.7) (p=0.31)	24.5 (24.4 – 26.3)	0.715
Septic Shock[d] (n)	7	18	0.975	-	-	
Source of infection (n)			0.810	-	-	
Pneumonia	3	12				
Abdominal	3	6				
Other/unknown	1	2				
Organism (n)			0.544	-	-	
Gram-positive bacteria	5	8				
Mixed	0	1				
Gram- negative	2	4				

bacteria						
Unknown	0	4				
Other	0	3				
Mechanical ventilation (days)	16 (11.2 – 23)	11.0 (6.5 – 21.0)	0.425	-	-	
Acute pulmonary injury (n) (ARDS)[e]	4	13	0.933	-	-	
				-	-	
AKIN score[f]			0.810	-	-	
No	4	12				
Stage I	1	1				
Stage II	0	1				
Stage III	2	6				
Apache II[g] score	28 (21.5 – 32.5)	31.0 (22.5 – 33.0)	0.343	-	-	
SOFA[h] score	16 (13 – 16)	13.0 (12.0 – 14.2)	0.087	-	-	
Serum lactate levels (mmol/l)	4.3 (3.5 -11.9)	3.2 (1.6 – 4.3)	0.135	-	-	
ICU length-of-stay (days)	25 (18 – 36.5)	15.5 (9.8 – 30.2)	0.151	-	-	
Hospital length-of-stay (days)	30 (22 – 51.5)	29.0 (16.0 – 38.5)	0.503	-	-	
Death (n)	4	3	0.091	-	-	

[a]indicates p-value for comparison between NGS and RT-qPCR group. Statistical test used: Kruskal-Wallis as non-parametric test and chi^2 for categorial comparison;

[b]p-values for comparison to the NGS group,
[c] p-value for comparison to healthy volunteers of the RNAseq cohort
[d]Septic shock was defined according to Sepsis-3 criteria (1)
[e]Acute Respiratory Distress Syndrome (defined according to Berlin criteria (2)
[f]Acute Kidney Injury Network Staging System for Acute Kidney Injury classification system with a range of 1 to 3 with higher scores indicating more severe injury (3)
[g]Acute Physiology and Chronic Health Evaluation II is a severity-of-disease classification system for critically ill patients. Scores range from 0 to 71, higher scores correspond to more severe disease and a higher risk of death (4)
[h]Sequential Organ Failure Assessment (SOFA) score is a scoring system to determine the extent of a person's organ function or rate of failure. Scores less than 9 indicate predictive mortality at 33% while above 11 can be close to or above 95% (5)

Table S2: Values of SOFA criteria used to calculate the ICU admission SOFA score in the RNAseq and the RT-qPCR confirmation group.

**SOFA - Criteria**	**Variable**	**Sepsis - RNAseq (n=7)**	**Sepsis – RT-qPCR confimation (n=20)**	**p-value**
Respiratory system	PaO2/FiO2	137.7 (118.7 - 162.5)	112.3 (90.6 - 142.0)	0.157
Nervous system	Glasgow Coma Scale	3.0 (3.0 - 3.0)	3.0 (3.0 - 10.2)	0.055
Cardiovascular system	Mean arterial pressure OR administration of vasopressors required			
Noradrenaline (µg/kgBW/min)		0.5 (0.3 - 0.8)	0.3 (0.1 - 0.7)	0.306
Vasopressin		0.0 (0.0 - 0.5)	0.0 (0.0 - 0.2)	1
MAP min (mmHg)		65.0 (62.0 - 70.0)	64.0 (55.0 - 68.5)	0.618

Liver	Bilirubin (mg/dl)	1.2 (1.2 - 3.2)	1.2 (0.5 - 2.4)	0.289
Coagulation	Platelets (x10³/ml	70.0 (56.5 - 133.0)	179.0 (123.5 - 282.0)	0.025
Kidneys	Creatinine (mg/dl)	1.2 (0.9 - 1.8)	1.4 (1.0 - 2.2)	0.643
**Admission SOFA score**		**16 (13 – 16)**	**13 (12 – 14)**	**0.087**

Table S3: Comparison of RNA characterizations between patients with sepsis and healthy volunteers.

	Sepsis ± SD	Volunteer ± SD	P value
**RNA concentration [ng/µl]**	231.71 ± 173.25	149.17 ± 44.90	0.28
**miRNA reads**	5.73E6 ± 2.49E6	6.34E6 ± 2.22E6	0.65
**mRNA reads**	4.62E6 ± 2.91E6	2.48E6 ± 5.58E5	0.11

SD: standard deviation

Table S4: Detection of unique miRNA and mRNA transcripts by RNA sequencing. Data represent transcripts that were detected with a mean per-group expression of at least 20 reads.

	Sepsis	Volunteer	Overlap
**miRNAs**	297	316	278 (82.99 %)
**mRNAs**	4,999	5,142	4,569 (82.00 %)

Table S5: Top 5 up- and downregulated miRNAs and mRNAs.

	**Transcript**	**baseMean**	**Log2FC**	**padj**
miRNA Down in patients	miR-10395-3p	415.46	-4.71	0.003
	miR-6803-3p	58.73	-2.72	<0.001
	miR-296-5p	59.31	-2.70	0.001
	miR-320d	88.19	-2.50	0.002
	miR-150-5p	4595.50	-2.43	<0.001
miRNA Up in patients	miR-10b-5p	341.79	3.63	<0.001
	miR-143-3p	1286.34	4.10	<0.001
	miR-199b-5p	174.58	4.45	<0.001
	miR-122-5p	1448.09	6.65	<0.001
	miR-582-3p	319.11	6.68	<0.001
mRNA Down in patients	FCER1A	32.30	-5.46	<0.001
	PACSIN1	13.69	-4.84	<0.001
	ZNF683	22.89	-4.71	<0.001
	LYPD2	20.43	-4.64	<0.001
	PTGDS	72.98	-4.40	<0.001
mRNA Up in patients	PCSK9	33.22	8.47	<0.001
	CD177	5496.72	8.75	<0.001
	CYP19A1	59.83	9.32	<0.001
	ADAMTS2	64.19	9.43	<0.001
	MMP8	2868.41	9.67	<0.001

baseMean: mean expression; log2FC: log2 fold change; padj: adjusted p-value

Table S6: Successfully validated miRNAs and mRNAs in the second patient cohort (biologic validation).

	NGS		qPCR Validation	
**Molecule**	**Log2FC**	**padj**	**Log2FC**	**p-value**
**miR-17-5p**	3.88	0.013	-1.10	0.081
**miR-30c-5p**	2.57	<0.001	0.82	<0.001
**miR-92a-3p**	-2.46	0.016	-0.68	0.005
**miR-486-5p**	-1.23	0.017	-0.88	<0.001
**let-7a-5p**	-0.60	0.448	-1.82	<0.001
**ACE**	-1.48	0.016	-2.01	0.011
**AKR1B1**	-1.56	<0.001	-1.50	0.003
**ANXA1**	2.32	<0.001	1.77	0.003
**BCL2**	-2.03	<0.001	-1.59	0.015
**IGFBP3**	-3.09	<0.001	-2.97	<0.001
**LCN2**	7.03	<0.001	2.96	0.021
**MMP9**	3.27	<0.001	1.79	0.023
**MYC**	-1.18	<0.001	-1.12	0.047
**PDE4B**	-1.17	0.015	-1.62	0.007
**PPARG**	3.65	<0.001	2.90	<0.001
**SMAD3**	-1.63	<0.001	-1.93	<0.001
**TP53**	-1.52	<0.001	-2.11	<0.001
**TSPO**	2.48	<0.001	1.30	0.003

Table S7: Significantly regulated molecules according to SOFA Cluster 1-6 from the RNAseq data set.

SOFA-Cluster	Molecule	log2FC	padj
1	miR-125b-5p	-1.529	0.010
1	miR-145-5p	1.757	0.020
1	miR-150-5p	-2.427	<0.001
1	miR-20a-5p	-1.972	0.014
1	miR-223-3p	1.592	0.008
1	miR-296-5p	-2.702	0.001
1	miR-374b-5p	1.757	0.035
1	ACE	-1.478	0.015
1	CTSG	7.056	<0.001
1	CYP51A1	1.225	0.007
1	ELANE	6.591	<0.001
1	F12	1.577	0.001
1	FLT1	1.768	0.005
1	FOXO3	1.322	0.010
1	GAPDH	1.650	<0.001
1	IGFBP3	-3.085	<0.001
1	IL18	1.072	0.006
1	LTA	-2.050	<0.001
1	LY96	2.505	<0.001
1	NAMPT	1.814	<0.001
1	PRTN3	7.108	<0.001
1	TSPO	2.484	<0.001
1	VEGFA	1.023	0.005

SOFA-Cluster	Molecule	log2FC	padj
2	let-7b-5p	-1.863	<0.001
2	miR-199b-5p	4.448	<0.001
2	miR-320a-3p	-1.209	0.003
2	miR-451a	1.335	0.006
2	ACHE	3.516	<0.001
2	ADA	-1.412	<0.001
2	CACNA2D2	-3.602	<0.001
2	CACNA2D3	-2.863	<0.001
2	ENO2	-2.393	<0.001
2	LCN2	7.025	<0.001
2	MMP9	3.269	<0.001
2	PDE4B	-1.167	0.015
2	PDE4D	1.566	0.001
2	PDE8A	-1.167	<0.001
2	PRTN3	7.108	<0.001
2	RTN4	1.395	<0.001
2	TIMP1	1.295	0.005

SOFA-Cluster	Molecule	log2FC	padj
3	let-7b-5p	-1.863	<0.001
3	miR-10b-5p	3.631	<0.001
3	miR-125b-5p	-1.529	0.010
3	miR-142-3p	1.825	0.002
3	miR-143-3p	4.104	<0.001
3	miR-145-5p	1.757	0.020
3	miR-150-5p	-2.427	<0.001
3	miR-20a-5p	-1.972	0.014
3	miR-192-5p	1.349	0.012
3	miR-194-5p	1.305	0.042
3	miR-199b-5p	4.448	<0.001
3	miR-24-3p	1.096	0.032
3	miR-26a-5p	1.235	0.043
3	miR-296-5p	-2.702	0.001
3	miR-320a-3p	-1.209	0.003
3	miR-324-3p	-1.925	0.020
3	miR-342-3p	-1.384	0.021
3	miR-374b-5p	1.757	0.035
3	miR-451a	1.335	0.006
3	miR-486-5p	-1.229	0.017
3	miR-27b-3p	1.114	0.049
3	ALOX5	1.665	<0.001
3	BCL2	-2.031	<0.001
3	CEBPB	1.707	<0.001
3	IL1RN	1.873	<0.001
3	MYC	-1.177	<0.001
3	NEDD4	1.301	<0.001
3	PPARG	3.647	<0.001
3	PTEN	1.194	<0.001
3	SMAD3	-1.628	<0.001
3	TGFBR1	1.260	<0.001
3	TP53	-1.520	<0.001
3	VEGFA	1.023	0.005
3	miR-30c-5p	2.566	<0.001
3	miR-92a-3p	-1.311	0.016
3	miR-99b-5p	1.455	0.005

2	TSPO	2.484	<0.001

SOFA-Cluster	Molecule	log2FC	padj
4a	miR-10b-5p	3.631	<0.001
4a	miR-125b-5p	-1.529	0.010
4a	miR-192-5p	1.349	0.012
4a	miR-24-3p	1.096	0.032
4a	miR-26a-5p	1.235	0.043
4a	miR-27b-3p	1.114	0.049
4a	miR-328-3p	-1.474	0.001
4a	miR-92a-3p	-1.311	0.016
4a	ACE	-1.478	0.015
4a	ADM	2.495	<0.001
4a	AKR1B1	-1.559	<0.001
4a	APRT	-1.384	<0.001
4a	CD44	1.148	0.001
4a	CXCR3	-3.237	<0.001
4a	ENTPD1	1.258	<0.001
4a	GAMT	-1.699	<0.001
4a	GRN	1.156	<0.001
4a	IL1R1	2.334	<0.001
4a	KL	3.699	<0.001
4a	PARP1	-1.318	<0.001
4a	SLC14A1	2.923	0.007
4a	SLC4A1	2.500	0.012
4a	SMAD3	-1.628	<0.001
4a	STAT4	-2.073	<0.001
4a	VAV2	-1.190	0.002
4b	let-7b-5p	-1.863	<0.001
4b	miR-10b-5p	3.631	<0.001
4b	miR-125b-5p	-1.529	0.010
4b	miR-145-5p	1.757	0.020
4b	miR-199b-5p	4.448	<0.001
4b	miR-20a-5p	-1.972	0.014
4b	miR-24-3p	1.096	0.032
4b	miR-26a-5p	1.235	0.043
4b	miR-320a-3p	-1.209	0.003
4b	miR-328-3p	-1.474	0.001
4b	miR-503-5p	1.389	0.002
4b	miR-92a-3p	-1.311	0.016
4b	ADM	2.495	<0.001
4b	ANXA1	2.318	<0.001
4b	AQP1	1.716	0.024
4b	CD44	1.148	0.001
4b	CDKN1A	1.021	0.039
4b	CLU	1.629	0.001

SOFA-Cluster	Molecule	log2FC	padj
5	let-7b-5p	-1.863	<0.001
5	miR-125b-5p	-1.529	0.010
5	miR-145-5p	1.757	0.020
5	miR-26a-5p	1.235	0.043
5	miR-328-3p	-1.474	0.001
5	miR-92a-3p	-1.311	0.016
5	ABCC2	1.363	0.011
5	ABCG2	2.625	0.019
5	EPAS1	2.483	<0.001
5	GALNT3	1.303	0.001
5	GFER	-1.105	<0.001
5	HGF	2.769	<0.001
5	OSM	2.093	<0.001
5	SCD	2.048	0.002
5	SLC51A	3.642	<0.001
5	SPTA1	3.686	<0.001
5	SPTB	2.543	0.005
5	TFRC	1.522	<0.001
5	TLR4	1.168	0.005
5	ZNF275	-1.489	<0.001

SOFA-Cluster	Molecule	log2FC	padj
6	miR-125b-5p	-1.529	0.010
6	miR-30c-5p	2.566	<0.001
6	miR-326	-1.273	0.020
6	BCL2	-2.031	<0.001
6	BCL2L1	2.037	0.021
6	CLEC11A	1.401	0.013
6	FLT1	1.768	0.005
6	FLT3LG	-2.295	<0.001
6	GATA1	1.871	0.035
6	IL4R	1.289	<0.001
6	ITGA2B	1.535	0.018
6	ITGAM	1.917	<0.001
6	JAK2	1.882	<0.001
6	let-7b-5p	-1.863	<0.001
6	miR-142-3p	1.825	0.002
6	miR-143-3p	4.104	<0.001
6	miR-150-5p	-2.427	<0.001
6	miR-20a-5p	-1.972	0.014
6	miR-296-5p	-2.702	0.001
6	miR-30a-3p	2.215	0.001
6	miR-320a-3p	-1.209	0.003

4b	FKBP1A	1.055	<0.001
4b	FOS	1.066	0.030
4b	GDF15	1.526	0.029
4b	HSPA1A/HSPA1B	1.458	<0.001
4b	LCN2	7.025	<0.001
4b	LGALS3	1.896	0.003
4b	MYC	-1.177	<0.001
4b	SPHK1	1.060	0.009
6	miR-92a-3p	-1.311	0.016
6	miR-451a	1.335	0.006
6	miR-486-5p	-1.229	0.017
6	NFE2	1.516	<0.001
6	NQO2	1.799	<0.001
6	PDGFB	-2.643	<0.001
6	PF4	1.502	0.023
6	THBS1	2.709	<0.001
6	TLR4	1.168	0.005

Table S8: Unique molecules related to each of the respective SOFA criteria. mRNAs confirmed by RT-qPCR in the second patient group are marked in red.

SOFA-Clusters						
**Hypoxia**	**Impaired GCS**	**Circulatory failure**	**Increase in Creatinine**	**Acute renal failure**	**Increase in Bilirubin**	**Low Platelet count**
miR-223-3p	ACHE	miR-194-5p	AKR1B1	miR-503-5p	ABCC2	miR-30a-3p
CTSG	ADA	miR-324-3p	APRT	ANXA1	ABCG2	IL4R
CYP51A1	ENO2	miR-342-3p	CXCR3	AQP1	EPAS1	ITGA2B
ELANE	MMP9	ALOX5	ENTPD1	CDKN1A	GALNT3	ITGAM
F12	PDE4B	ANGPT2	GAMT	CLU	GFER	JAK2
FOXO3	PDE4D	CEBPB	GRN	FKBP1A	HGF	BCL2L1
GAPDH	PDE8A	NEDD4	KL	FOS	OSM	CLEC11A
IGFBP3	RTN4	PPARG	PARP1	GDF15	SCD	FLT3LG
IL18	TIMP1	PTEN	SLC14A1	HSPA1/2	SLC51A	GATA1
LTA		TGFBR1	SLC4A1	LGALS3	SPTA1	NFE2
LY96		TP53	STAT4	SPHK1	SPTB	NQO2
NAMPT			VAV2		TFRC	PDGFB
					ZNF275	PF4
						THBS1

Additional References

1. Hermann S, Buschmann D, Kirchner B, et al. Transcriptomic profiling of cell-free and vesicular microRNAs from matched arterial and venous sera. J Extracell Vesicles 2019;8(1):1670935.
2. Buschmann D, Kirchner B, Hermann S, et al. Evaluation of serum extracellular vesicle isolation methods for profiling miRNAs by next-generation sequencing. J Extracell Vesicles 2018;7(1):1481321.
3. Hu J, Ge H, Newman M, et al. OSA: a fast and accurate alignment tool for RNA-Seq. Bioinformatics 2012;28(14):1933-1934.
4. Love MI, Huber W, Anders S. Moderated estimation of fold change and dispersion for RNA-seq data with DESeq2. Genome Biol 2014;15(12):550.
5. Kramer A, Green J, Pollard J, Jr., et al. Causal analysis approaches in Ingenuity Pathway Analysis. Bioinformatics 2014;30(4):523-530.
6. Lambden S, Laterre PF, Levy MM, et al. The SOFA score-development, utility and challenges of accurate assessment in clinical trials. Crit Care 2019;23(1):374.
7. Andersen CL, Jensen JL, Orntoft TF. Normalization of real-time quantitative reverse transcription-PCR data: a model-based variance estimation approach to identify genes suited for normalization, applied to bladder and colon cancer data sets. Cancer Res 2004;64(15):5245-5250.
8. Reithmair M, Buschmann D, Marte M, et al. Cellular and extracellular miRNAs are blood-compartment-specific diagnostic targets in sepsis. J Cell Mol Med 2017;21(10):2403-2411.
